# *Mex3c* mutation affects lactation through impairing milk ejection in female mice

**DOI:** 10.1042/BSR20201285

**Published:** 2020-12-10

**Authors:** Yong Du, Dongjun Sun, Yan Li

**Affiliations:** 1 Department of Surgical Research, General Hospital, Ningxia Medical University, Ningxia 750004, China; 2 Graduate School, Ningxia Medical University, Ningxia 750004, China; 3 Institute for Regenerative Medicine, Wake Forest University Health Sciences, Winston-Salem, NC 27157, U.S.A.; 4 Department of Obstetrics and Gynecology, General Hospital, Ningxia Medical University, Ningxia 750004, China

**Keywords:** gene trap mouse, lactation, mammary gland, MEX3C

## Abstract

Mouse *Mex3c* encodes RNA-binding proteins of variant length through alternative splicing. Its mutation results in multiple defects including growth retardation, perturbed energy balance, and defective antiviral innate immunity. Here we report that *Mex3c* mutation affects mammary gland development and lactation in female mice. Pups of *Mex3c* mutant dams die of starvation soon after birth. Milk contents are present in the alveoli but deficient in the ducts of the mammary glands in mutant mice. Mutant mice do not show prolactin or oxytocin deficiency. They also develop myoepithelial cells in the mammary glands. *Mex3c* is expressed in the mammary gland epithelium. Our data suggest that functional defects in mammary gland epithelium or myoepithelial cells could cause lactation defects.

## Introduction

During puberty, the quiescent mammary epithelium begins a branching morphogenic process controlled by hormones and other factors, and develops an arboreal structure composed of apically oriented luminal epithelial cells that are surrounded by contractile myoepithelial cells. During pregnancy, the alveolar epithelium proliferates under the control of hormones and develops alveoli capable of producing milk. During lactation, the prolactin stimulates the luminal epithelial cells to produce and secrete milk proteins into the lumen of the alveoli, and oxytocin stimulates the contraction of the surrounding myoepithelial cells to move the milk through the ductal tree to the nipple [1].

Transgenic mice are useful models for better understanding the roles of genes involved in milk secretion. Various gene defects can impair milk secretion. For example, knocking out the *Mkl1* gene or overexpressing human protein C gene impairs development of the mammary gland and milk secretion [2–4]. Knocking out α-lactalbumin or overexpressing *Akt1* affects viscosity of the milk and impairs milk secretion [5,6]. Knocking out the oxytocin gene or its receptor impairs milk secretion due to defects in the milk ejection reflex [7–9]. Nevertheless, the signaling transduction pathways controlling milk secretion in the mammary gland and the functions of many genes involved in the process are not completely known.

In mice, the *Mex3c* gene encodes an RNA-binding ubiquitin E3 ligase, MEX3C, which has two hnRNP K homology (KH) domains binding to single-stranded RNA [10], and a C-terminal ring finger domain with ubiquitin E3 ligase activity [11,12]. At least three MEX3C protein variants can be expressed by alternative splicing and alternative transcription initiation: MEX3C-1 (the longest variant, previously termed as MEX3C^652AA^), MEX3C-2 (MEX3C^464AA^) and MEX3C-3 (MEX3C^372AA^) [13,14]. MEX3C-1 shuttles between the nucleus and the cytoplasm to adapt exportin 1-mediated *FOS* mRNA nuclear export [13]. It transfers K63-linked ubiquitin chains to RIG-I (DDX58) to enhance RIG-I-mediated antiviral innate immunity [12], and to CNOT7 to facilitate degradation of *MHC-I* mRNA [15]. MEX3C-1, -2 and -3 all interact with adaptor protein-related complex 2 (AP-2) and play a role in exosomal secretion of miR-451a [14]. In humans, *MEX3C* contributes to genetic susceptibility to hypertension [16] and represses chromosomal instability of tumor cells [17]. In mice, *Mex3c* is involved in regulating postnatal growth [18] and energy expenditure [19,20], although the mechanisms remain unknown.

While breeding *Mex3c* mutant females to generate mutant mice, we unexpectedly observed that most pups born to *Mex3c* mutant females could not thrive. Here we show that although the mutant females developed mammary glands and could produce milk, they appear unable to eject their milk. It is likely that MEX3C proteins regulate the expression of some important components in the oxytocin/oxytocin receptor signaling pathway. Further work will determine the molecular mechanisms for *Mex3c*’s involvement in lactation.

## Materials and methods

### Animals

The *Mex3c* gene trap allele *Mex3c^Gt(DD0642)Wtsi^* (simplified as ‘*tr*’, indicating the trapped allele) was described earlier [18], which had been backcrossed at least five generations to a mainly FVB/N background. Experiments were conducted in accordance with the National Research Council publication *Guide for Care and Use of Laboratory Animals* and approved by the Institutional Animal Care and Use Committee of Wake Forest University Health Sciences. Mouse genotyping was performed using lysates of ear biopsies, as previously described [18,21].

### Generation of *Mex3c* gene trap mice

The *Mex3c* gene trap ES cell line DD0642 was obtained from the Sanger Institute Gene Trap Resource (SIGTR, Cambridge, U.K.). The ES cells were microinjected into mouse blastocysts, and the resulting chimera males were mated with C57/BL6 females to obtain heterozygous Mex3c gene trap mice. Heterozygous mice were intercrossed to obtain mutant mice of 129Sv/C57 mixed background. Heterozygous mice were backcrossed to C57/BL6 or FVB/N for six generations to obtain mutant mice of mainly C57/BL6 or FVB/N background. Mice were housed in the animal facility of Wake Forest University Health Sciences (Winston-Salem, NC). Experiments were conducted in accordance with the National Research Council Publication Guide for the Care and Use of Laboratory Animals, and were approved by the Institutional Animal Care and Use Committee of Wake Forest University with approval No. A14-014. Mice were kept in microisolator cages with 12-h light/dark cycles and were fed *ad libitum*. A chow diet (Prolab, RMH3000; PMI Nutrition International, Henderson, CO) was used for colony maintenance. Mice were subjected to CO_2_ asphyxiation and apnea was confirmed. They were then subjected to cervical dislocation or removal of the lungs for secondary assurances.

### Blood hormone assays

Serum was obtained from the saphenous vein of adult virgin mice. Mouse prolactin was assayed with a kit from Thermo Fisher Scientific (Cat.# EMPRL). Mouse serum oxytocin was assayed with an ELISA kit from Enzo Life Sciences (Cat.# ADI-900-153A). Before detecting oxytocin, sera from two to three mice were combined and extracted with a Sep Pak C18 column (Waters, Milford, MA) according to the instructions of the manufacturer.

### Mammary gland whole-mount X-gal staining

To examine the expression of *Mex3c* in the mammary gland through detecting β-galactosidase (β-gal) activity, the fourth mammary glands of wildtype or heterozygous *Mex3c* gene trap mice were dissected and stained with X-Gal as described [22]. Some mammary glands stained with X-Gal were then stained with Carmine alum to visualize the mammary gland epithelium. Stained tissues were then embedded in paraffin and sectioned to observe under the microscope. Images were taken with an Axio M1 microscope equipped with an AxioCam MRc digital camera (Carl Zeiss).

### Mammary gland tissue histology and immunostaining

The fourth mammary glands were dissected and fixed in 4% paraformaldehyde at 4°C overnight for cryosections and paraffin sections. Five to eight micrometer paraffin sections were stained with Hematoxylin and Eosin.

For immunostaining, the paraffin sections were antigen-retrieved by steaming. Then the sections were blocked in 5% FBS and incubated with primary antibodies at room temperature for 1 h. Primary antibodies used included rabbit anti-perilipin 2 (PLIN2) antibody (Thermo Fisher Scientific, Cat#: PA1-16972, 1:200) and mouse anti-smooth muscle α actin (Santa Cruz Biotechnology, Cat#: sc32251, 1:300). After three washes in PBS buffer, they were incubated in Texas-red conjugated secondary antibodies (Vector, 1:500) for 1 h. After three washes in PBS, the sections were mounted with DAPI-containing mounting medium (Vector).

### Statistical analysis

We performed two-tailed Student’s *t* tests to compare differences between two groups. Tukey’s post-tests following analysis of variance (ANOVA) was performed for comparisons among the groups. *P*-value <0.05 was regarded as statistically significant.

## Results

### Pups born to mutant females could not thrive

We generated a *Mex3c* mutant mouse line by gene trapping and studied its roles in postnatal growth and energy expenditure [18–20]. When we attempted to use homozygous *Mex3c* gene trap females (*Mex3c^tr/tr^*, ‘tr’ indicates the trapped allele) to generate mutant mice, we observed that pups born to such mice were alive and appeared normal right after birth, but most died within 24 h regardless of genotypes. This was not observed in pups born to heterozygous females (*Mex3c^+/tr^*). We noted that on the second day after delivery, four mutant females lost all of their pups and two mutant females had only one or two pups left alive. Since all dead pups lacked milk in their stomachs, they apparently died from starvation. When pups from mutant females were fostered by a normal lactating dam, they thrived. We also observed that the mutant females attempted to care for their pups. Thus, the defects were most likely associated with milk production or ejection, rather than nursing behavior.

### *Mex3c* mutant females showed delayed mammary gland development

**Figure 1:**
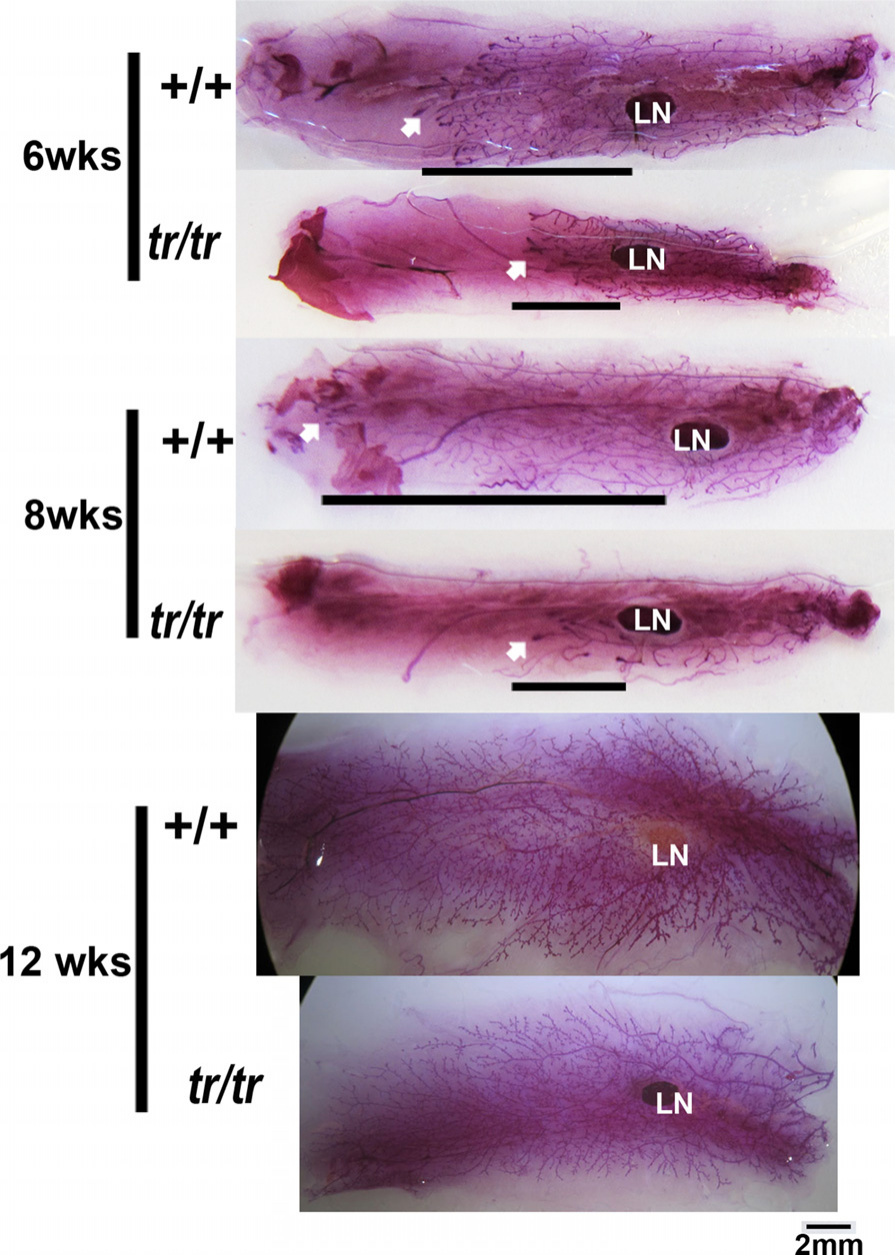
*Mex3c* mutation delays mammary gland development Entire inguinal mammary glands were collected at different ages. The control and mutant mice were littermates and had been housed in the same cages. Whole-mount Carmine staining was performed. Horizontal lines indicate the distance from the mammary gland origin to the front of the terminal end buds (white arrows). Abbreviation: LN, lymph node.

Previously we reported that *Mex3c* mutation caused local bone IGF1 deficiency [18]. Others had reported that local, but not circulating, IGF1 was important for mammary gland development [23]. Our observation of pup starvation prompted us to examine the development of mammary glands of mutant females. We compared whole-mount inguinal mammary glands (no. 4) isolated from age-matched control and mutant females housed in the same cages. At 6 and 8 weeks after birth (three pairs compared at each age), the terminal end buds of control females extended further beyond the lymph nodes than those of mutant females (Figure 1). However, at 12 weeks, the mammary glands of all four mutant mice examined reached the edge of the fat pad, although the fat pad of mutant mice was smaller than those of control mice, consistent with reduced adiposity in the mutant mice [19]. Our observations suggest that *Mex3c* mutation delayed, but did not abolish mammary gland development.

### *Mex3c* mutant mice did not show prolactin or oxytocin deficiency

To examine whether *Mex3c* mutation might impair the expression of prolactin and oxytocin—the hormones responsible for milk production [24] and ejection [7–9], respectively—we compared the plasma concentrations of the two hormones in control and mutant adult mice. Neither hormone showed deficiency in the mutants (Table 1). Indeed, mutant females had higher prolactin concentration than control females. Since virgin mutant mice did not show the deficiency of the two hormones, it is unlikely that they have reduced levels of those hormones during lactation.

**Table 1:** Plasma prolactin and oxytocin concentrations of control and *Mex3c* mutant mice

	Male		Female	
	Control	*Mex3c^tr/tr^*	Control	*Mex3c^tr/tr^*
**Prolactin (ng/ml)**	11.2 ± 6.8 (5)	21.6 ± 8.7 (5)	10.2 ± 4.2 (5)	115.6 ± 42.4 (5)*
**Oxytocin (ng/ml)**	0.16 ± 0.01 (9)	0.24 ± 0.06 (5)	0.19 ± 0.05 (4)	0.25 ± 0.04 (3)

* mutant females had higher prolactin concentration than control females.

For prolactin assay, serum from individual mouse was assayed. For oxytocin, serum from two to three mice of the same genotype and sex were pooled to obtain enough serum for the assay. The numbers in the parentheses are *n* of mice (for prolactin) or pooled groups (for oxytocin). *, *P*<0.05 when prolactin of control females was compared with that of mutant females (ANOVA). The rest of the parameters did not show a significant difference between control and mutants (*P*>0.05).

### *Mex3c* mutant mice produced milk after delivery of pups

**Figure 2:**
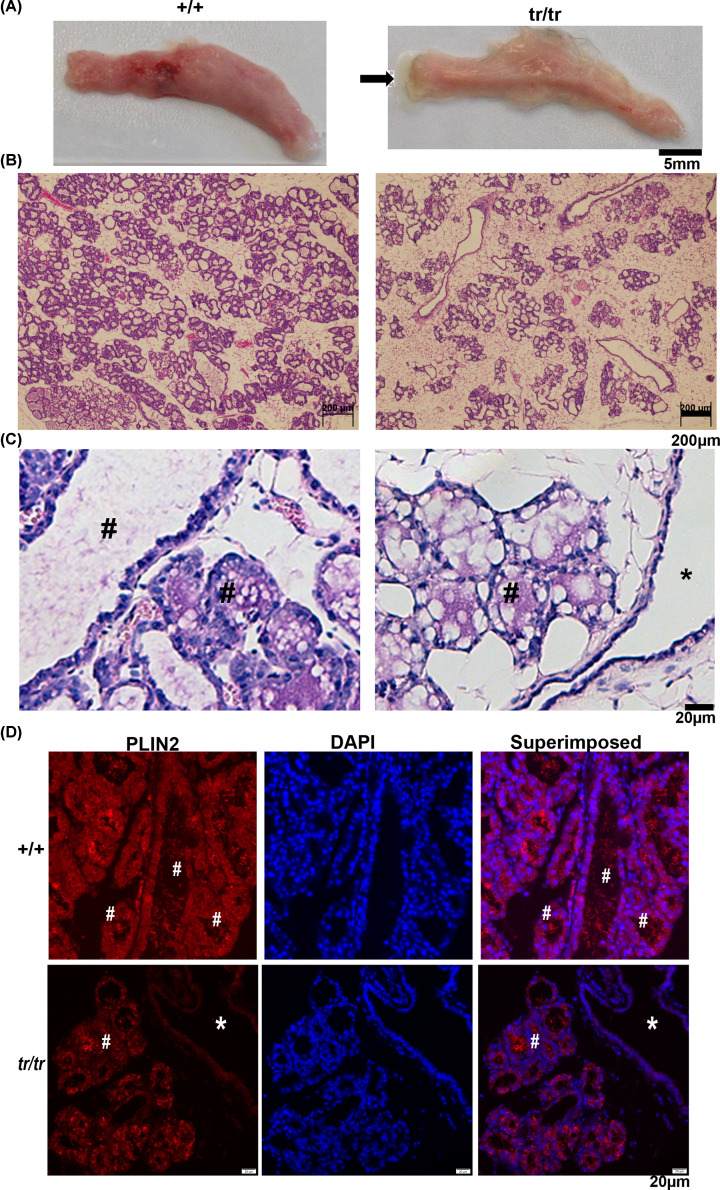
Analyses of mammary gland on lactation day 2 (**A**) Morphology of mammary glands on lactation day 2. The fourth mammary glands were dissected. Milk in the mammary glands of mutant mice was visible (arrowhead). (**B**) Reduced epithelial density of mammary glands of mutant mice on lactation day 2 (Hematoxylin and Eosin staining). (**C**) Milk content was not observed in the mammary gland ducts of mutant mice. Milk contents were marked by #. Ducts in mutant mice without milk contents marked by *. (**D**) Immunostaining for the milk protein, PLIN2.

To determine why the mutant females could not nurse their pups, we examined the mammary glands of pregnant females. On lactation day 2, the mammary glands of pregnant female mutants were indistinguishable in size from those of normal control mice (three pairs compared), possibly due to the stimulation of hormones during pregnancy. While collecting the mammary glands on lactation day 2, we observed plenty of milk in the mammary glands of the mutant females (Figure 2A, indicated by an arrow). However, histologic examination showed that mammary gland alveoli of the mutant mice were less dense (Figure 2B). Possible causes include the consequences of *Mex3c* mutation on mammary gland development and the involution of the mammary gland due to the inability to deliver the milk. In addition, milk contents were present in the alveoli and the ducts in control mice, but only in the alveoli (indicated by #) in mutant mice (Figure 2C, * marks a duct without milk content).

We then stained the mammary gland sections with an antibody against PLIN2 (ADFP), a protein associated with the surface of lipid droplets that is abundant in milk [25]. In both groups of mice, the mammary gland epithelium was positive for this protein (Figure 2D). However, in mutant mice, PLIN2 was only visible in the alveoli (indicated by #; * marks a duct negative for PLIN2). Thus, although mutant mice could produce milk, the process of moving their milk from the alveoli to the ducts was defective.

**Figure 3:**
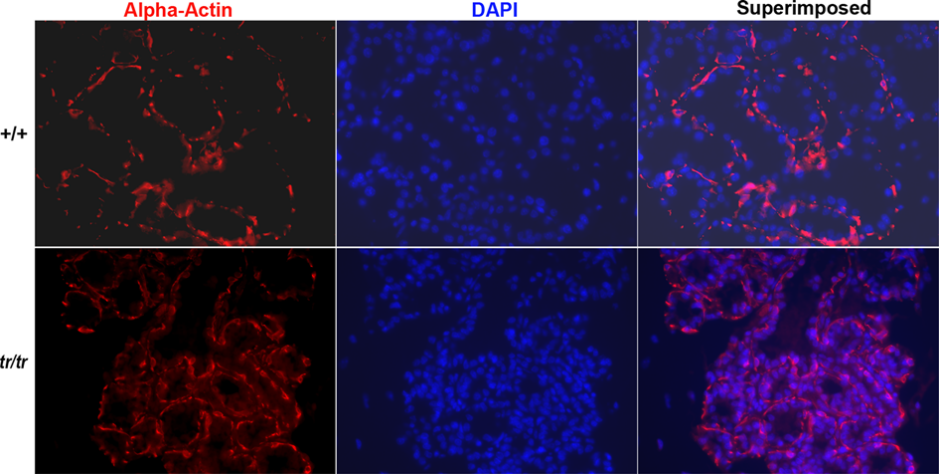
Myoepithelial cells in the mammary glands at lactation day 2 Anti-smooth muscle α-actin antibody was used to stain cryosections of mammary glands. Nuclei were counterstained by DAPI.

Since the contraction of the myoepithelial cells around the alveolar cells moves the milk from the alveoli to the ducts, we wondered whether the mutant mice have myoepithelial cells in their mammary glands. We immunostained mammary glands of lactation day 2 for myoepithelial cells with an anti-smooth muscle α-actin antibody. No myoepithelial cell deficiencies were seen in mutants compared with controls (Figure 3). Thus, the inability of mutant mice to eject milk was not caused by the lack of myoepithelial cells, but most likely by functional defects of myoepithelial cells or mammary gland epithelial cells.

### *Mex3c* is expressed in the mammary gland epithelium

**Figure 4:**
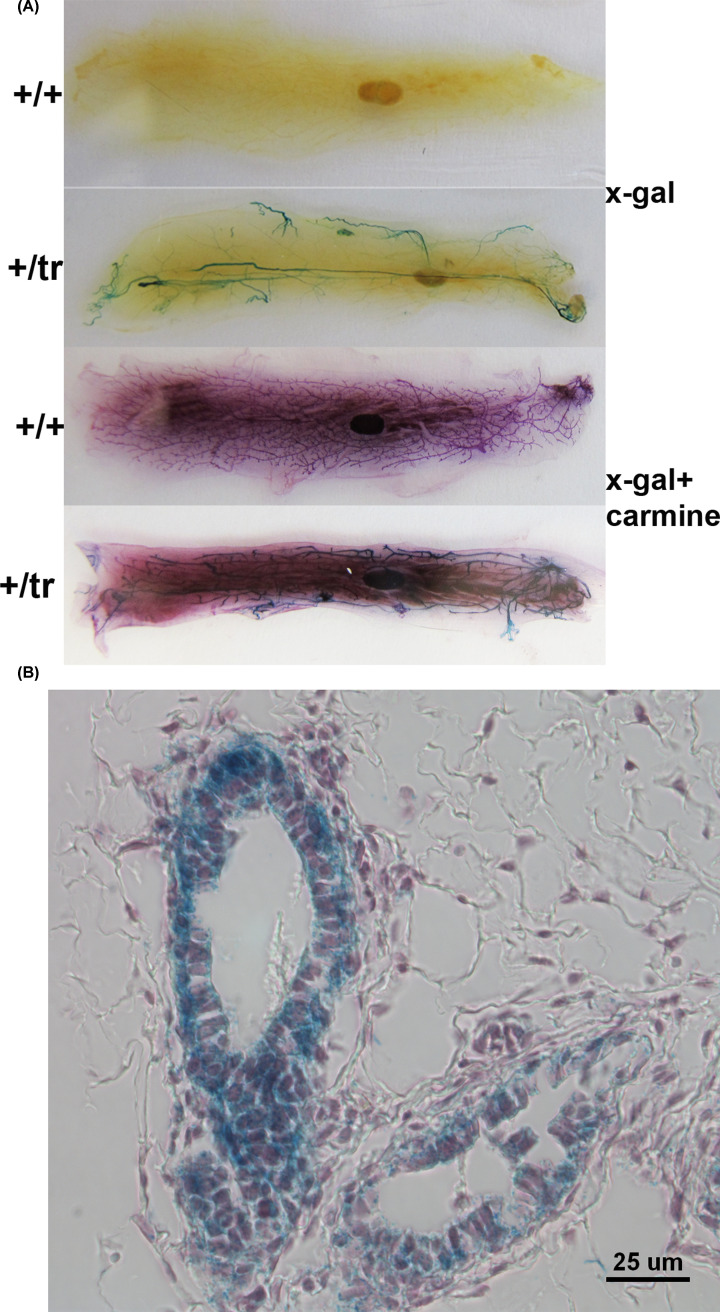
Expression of Mex3c in the mammary gland (**A**) Whole-mount staining of mammary glands by X-gal. Mammary glands were stained with (bottom two images) or without (top two images) Carmine alum after X-gal staining. (**B**) Sections of the double-stained mammary glands. Blue color indicates *Mex3c* expression.

To explore whether defects outside of the mammary gland occurred in mutant mice, we examined *Mex3c* expression in the mammary glands. In our *Mex3c* gene trap mice, gene trapping caused the expression of a *Mex3c-LacZ* fusion mRNA, whose transcription was driven by the endogenous *Mex3c* promoter. β-gal, the gene product of *lacZ*, reflects the expression of *Mex3c* expression [18]. We stained mammary glands of control and heterozygous mice with X-gal to examine *Mex3c* expression. On whole-mount staining, mammary gland ductal trees of the heterozygotes (+/tr), but not the homozygous wildtype mice, stained blue (Figure 4A). This result suggested that *Mex3c* mRNA was expressed in the mammalian gland. On mammary gland sections, epithelial cells were clearly positive for β-gal (Figure 4B). Thus, *Mex3c* is transcribed in the mammary gland epithelial cells. We were unable to tell whether myoepithelial cells express *Mex3c* due to difficulties in finding specific anti-smooth muscle α-actin antibody and anti-β gal antibody suitable for double immunostaining of the mouse mammary glands.

## Discussion

Here we describe lactation defects observed in *Mex3c* mutant female mice. The observation that pups of mutant females survived when nursed by normal lactating females suggests that the defects are in the mutant dams, not the pups. Mutant females can produce milk but appear unable to move the milk to the nipples. *Mex3c* is expressed in the mammary gland. Although *Mex3c* mutation delays mammary gland development in the mutants, possibly due to reduced IGF1 expression, lactation defects are most likely the result of functional defects of the mammary gland epithelium or myoepithelial cells, rather than the developmental or structural defects of the mammary glands.

How *Mex3c* mutation causes the lactation defects is unclear and warrants further study. *Mex3c* encodes MEX3C-1 (the longest protein), MEX3C-2 (the protein with 464 AA) and MEX3C-3 (the protein with 372 AA) [13,14]. All have a ring finger domain at the C-termini which functions as a ubiquitous E3 ligase [12,15,26]. Ubiquitination plays an important role in endocytosis and vesicle trafficking pathways [27]. We surmise that MEX3C deficiency likely impaired the milk secretion in the mammary glands through ubiquitination of important players in vesicle trafficking. We recently observed that MEX3C proteins interact with AP-2 [14], which plays an important role in endocytosis and vesicle trafficking [28,29]. The effects of AP-2 deficiency on lactation are unknown since AP-2 deficiency causes embryonic lethality [30]. Nevertheless, ubiquitin E3 ligase activity of MEX3C proteins or their interactions with AP-2 may underlie the lactation defects in *Mex3c* mutant females.

MEX3C proteins have at least one KH RNA-binding domain. We recently found that MEX3C-1 functions as an adaptor, facilitating the nuclear export of *FOS* mRNA [13]. Thus, it is possible that MEX3C regulates the stability or translation of some mRNAs, whose protein products are involved in milk secretion or ejection. The lactation defects in *Mex3c* mutant mice are similar to those observed in oxytocin- or oxytocin receptor-deficient mice [7–9]. It is likely that MEX3C proteins regulate the expression of some important components in the oxytocin/oxytocin receptor signaling pathway. Further work will determine the molecular mechanisms for *Mex3c*’s involvement in lactation.

## Abbreviations

ANOVA: analysis of variance
AP-2: adaptor protein-related complex 2
DAPI: 4′,6-diamidino-2-phenylindole
hnRNP: heterogeneous nuclear ribonucleoprotein
IGF1: insulin-like growth factor 1
KH: K homology
PLIN2: perilipin 2
β-gal: β-galactosidase
